# Utilizing Radiomics of Peri‐Lesional Edema in T2‐FLAIR Subtraction Digital Images to Distinguish High‐Grade Glial Tumors From Brain Metastasis

**DOI:** 10.1002/jmri.29572

**Published:** 2024-09-10

**Authors:** Emin Demirel, Okan Dilek

**Affiliations:** ^1^ Department of Radiology Faculty of Medicine, Afyonkarahisar University of Health Sciences Afyonkarahisar Turkey; ^2^ Department of Radiology Adana City Training and Research Hospital, University of Health Sciences Adana Turkey

**Keywords:** T2 WI, glioblastoma, brain metastasis, FLAIR WI, machine learning

## Abstract

**Background:**

Differentiating high‐grade glioma (HGG) and isolated brain metastasis (BM) is important for determining appropriate treatment. Radiomics, utilizing quantitative imaging features, offers the potential for improved diagnostic accuracy in this context.

**Purpose:**

To differentiate high‐grade (grade 4) glioma and BM using machine learning models from radiomics data obtained from T2‐FLAIR digital subtraction images and the peritumoral edema area.

**Study Type:**

Retrospective.

**Population:**

The study included 1287 patients. Of these, 602 were male and 685 were female. Of the 788 HGG patients included in the study, 702 had solitary masses. Of the 499 BM patients included in the study, 112 had solitary masses. Initially, the model was developed and tested on solitary masses. Subsequently, the model was developed and tested separately for all patients (solitary and multiple masses).

**Field Strength/Sequence:**

Axial T2‐weighted fast spin‐echo sequence (T2WI) and T2‐weighted fluid‐attenuated inversion recovery sequence (T2‐FLAIR), using 1.5‐T and 3.0‐T scanners.

**Assessment:**

Radiomic features were extracted from digitally subtracted T2‐FLAIR images in the area of peritumoral edema. The maximum relevance‐minimum redundancy (mRMR) method was then used for dimensionality reduction. The naive Bayes algorithm was used in model development. The interpretability of the model was explored using SHapley Additive exPlanations (SHAP).

**Statistical Tests:**

Chi‐square test, one‐way analysis of variance, and Kruskal–Wallis test were performed. The *P* values <0.05 were considered statistically significant. The performance metrics include area under curve (AUC), sensitivity (SENS), and specificity (SPEC).

**Results:**

The mean age of HGG patients was 61.4 ± 13.2 years and 61.7 ± 12.2 years for BM patients. In the external validation cohort, the model achieved AUC: 0.991, SENS: 0.983, and SPEC: 0.922. The external cohort results for patients with solitary lesions were AUC: 0.987, SENS: 0.950, and SPEC: 0.922.

**Data Conclusion:**

The artificial intelligence model, developed with radiomics data from the peritumoral edema area in T2‐FLAIR digital subtraction images, might be able to differentiate isolated BM from HGG.

**Evidence Level:**

3

**Technical Efficacy:**

Stage 2

The most common malignant lesions seen in the brain in adulthood are brain metastasis (BM) and high‐grade gliomas (HGGs).^1^, ^2^ With the World Health Organization (WHO) 2021 classification, the definition of glioblastoma (GBM), which previously included all grade 4 tumors, was changed to define only IDH‐wild grade 4 astrocytomas. IDH mutant grade 4 astrocytomas are excluded from this definition.^3^ Our study defined grade 4 IDH‐wild glioblastomas and IDH mutant grade 4 astrocytomas as HGG. The distinction between HGG and isolated BM has diagnostic, therapeutic, and prognostic implications. However, distinguishing between isolated brain metastases and primary tumors can be challenging. Histopathologic tissue evaluation is the reference standard. However, this is only occasionally possible, and complications related to the procedure may develop.^4^

On conventional imaging, factors such as lesions' multiplicity, morphology, cerebellar localization, and known history of the underlying primary cancer may help differentiate BM from GBM.^2^ However, brain metastases may occur as solitary lesions in approximately half of patients or may be associated with undiagnosed systemic malignancy in approximately 15%–30%.^5^ Therefore, conventional imaging alone may be insufficient for accurate classification.^2^, ^6^ Thanks to the innovations in MR imaging techniques in recent years, studies are increasing daily to determine methods to differentiate these two lesions from each other.^7^, ^8^ More advanced MRI techniques such as spectroscopy, perfusion, diffusion‐weighted, diffusion tensor imaging, and amide proton transfer imaging, which include the area of peritumoral edema, have been used to differentiate HGG from BM metastases.^9^, ^10^, ^11^ However, the long acquisition times, lengthy post‐processing procedures, the fact that the modalities are only actively available in some available devices, and the fact that they can be added to the devices at very high prices make applying these modalities to every case difficult. In addition, with the developments in artificial intelligence in recent years, radiomics data obtained from conventional or advanced MR imaging methods have been used to differentiate HGG tumors from BM.^12^, ^13^

Tumor angiogenesis with disruption of the blood–brain barrier is responsible for the vasogenic edema encountered in both benign and malignant brain lesions. Peritumoral edema develops in brain metastases with HGG. This area of peritumoral edema is similar in both cases on conventional brain MR imaging.

Radiomics can be defined as a system that extracts high‐throughput quantitative features from radiographic images, providing quantitative data far beyond what the eye can discern.^14^ With radiomics, it is possible to extract more quantitative features from traditional routine sequences than can be distinguished conventionally. Radiomic‐based biomarkers can provide crucial information in the diagnosis, classification, and therapeutic treatment of various solid tumors. Moreover, it is beginning to have an impact on the management of neuro‐oncological diseases, including low‐grade gliomas, high‐grade gliomas, and brain metastases.^15^ Research in this field encompasses a broad spectrum, including accurate classification of brain lesions, treatment planning, and assessment of treatment‐related changes.

It is not possible to visually evaluate the content of peritumoral edema in BM with HGG except that it is hyperintense in T2 and FLAIR images. However, previous studies have shown that glioblastoma and BM peritumoral edema contain differences in microstructural structure.^16^, ^17^ However, there are difficulties in applying these studies to large populations and variability in the success rates obtained. Patel et al^18^ demonstrated the differentiation of non‐contrasting low‐grade glial tumors from IDH mutant 1p19q codeletion patients with T2‐FLAIR mismatch sign by a noninvasive method. Cho et al^19^ showed that IDH mutant and non‐deleted low‐grade glioma patients could be predicted with high specificity but low sensitivity using the quantitative T2‐FLAIR mismatch signal and T2‐FLAIR digital subtracted images. Similar to non‐contrast‐enhancing low‐grade gliomas, the area of peritumoral edema is an area generally associated with the tumor but not contrast‐enhanced. We think there is a need for a quantitative method with a high success rate that can distinguish between HGG and BM. We aimed to distinguish high‐grade (grade‐4) glial tumors from solitary brain metastases using machine models developed based on radiomic data obtained from the perilesional edema area in T2‐FLAIR digital subtraction images created by digitally subtracting FLAIR‐weighted images from conventional T2‐weighted images.

## Materials and Methods

### Patients' Selection

All datasets did not contain any personal identifying information, and ethics committee approval and consent were obtained for the reference study of the open‐source dataset. We used a large number of open‐source databases in the study. High‐grade glial tumor cases from the multi‐parametric magnetic resonance imaging (mpMRI) scans for de novo Glioblastoma (GBM) patients from the University of Pennsylvania Health System (UPENN‐GBM) dataset were included in the training cohort.^20^ The University of California San Francisco, Brain Metastases Stereotactic Radiosurgery (UCSF‐BMSR) MRI dataset^21^ patients in the dataset to the training cohort. Some of the patients in the University of California San Francisco Preoperative Diffuse Glioma MRI (UCSF‐PDGM) dataset^22^ were selected for the validation cohort. BM patients in the Pretreat‐MetsToBrain‐Masks dataset were selected for the validation cohort, and patients in the TCGA‐GBM dataset^23^ were selected for the validation cohort. UPEN‐GBM, Pretreat‐MetsToBrain‐Masks, UCSF‐PDGM, and TCGA‐GBM from Cancer Imaging Archive (TCIA).^24^ The images of the patients in our datasets had undergone specific preprocessing procedures. Four standard conventional sequences were coregistered to the SRI24 anatomical template, resampled to a uniform isotropic resolution (1 mm^3^), and skull stripped. In the majority of patients, the mass was segmented using various methods into contrast‐enhanced area, necrosis, and peri‐lesional edema area, and these data were shared as open access. Patient characteristics, including age, sex, pathological grade, and genomic profile, were obtained from TCIA.

The inclusion criteria were as follows: Patients with grade 4 IDH mutant astrocytoma and grade 4 IDH wild‐type glioblastoma and patients with brain metastases without any history of treatment and surgery before imaging. The exclusion criteria were as follows: Patients for whom three regions of the mass (contrasted area, necrosis, peritumoral edema) were not automatically segmented, patients for whom IDH mutation data could not be reached in glial tumors, patients for whom no peritumoral edema area was detected.

**FIGURE 1:**
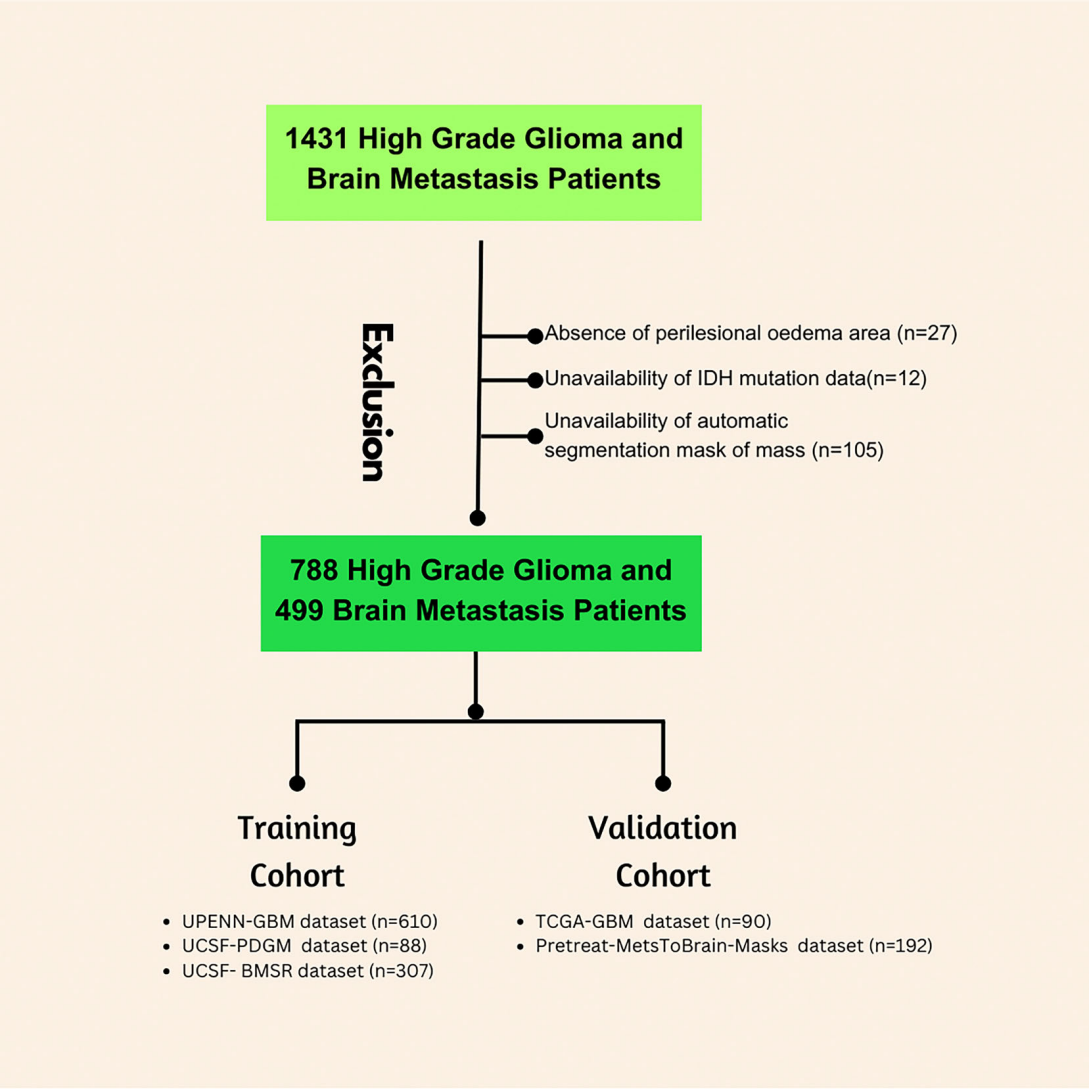
The number of patients included and excluded in the study and the number of train and validation cohorts are shown.

We found 1431 records that met the inclusion criteria. Of these, 105 were excluded because there was no automatic segmentation data of the mass, 12 patients were excluded because IDH mutation data were unavailable, 27 were excluded because there was no peritumoral edema area. As a result, 610 patients with high‐grade glial tumors from the UPENN‐GBM dataset, 88 patients with high‐grade glial tumors from the UCSF‐PDGM dataset, and 307 patients with brain metastases from UCSF—the BMSR dataset were included in the training cohort. The validation cohort included 90 patients with high‐grade glial tumors in the TCGA‐GBM database and 192 patients with brain metastases in the Pretreat‐MetsToBrain‐Masks database. A total of 1287 patients were included in the study (Fig. 1).

The study included 788 patients with high‐grade glial tumors. Of these, 47 were IDH mutant grade 4 astrocytoma patients, and 741 were grade 4 glioblastoma patients. Four hundred ninety‐nine patients with metastatic brain tumors were included. When the primary cancers of metastatic patients were analyzed, it was found that 229 patients had lung cancer, 99 patients had breast cancer, 91 patients had malignant melanoma, 29 patients had renal cell carcinoma, 23 patients had gastrointestinal cancers, and the remaining 28 patients had carcinoma of various other primary causes.

### Creation of T2‐FLAIR Digital Subtraction Images

In the datasets we utilized, pre‐processing steps including signal normalization, resampling, co‐registration, and skull stripping had already been performed on all four fundamental sequences (T1, T1 + C, T2, FLAIR). To digitally subtract FLAIR images from MR images, we first installed the necessary libraries (nibabel, numpy). T2 and FLAIR images were converted into numpy arrays, and the FLAIR image was mathematically subtracted from the T2 image. The resulting difference image was normalized; for this purpose, the image data were scaled between the minimum and maximum values. The normalized image was saved in NIfTI format and visualized.

**FIGURE 2:**
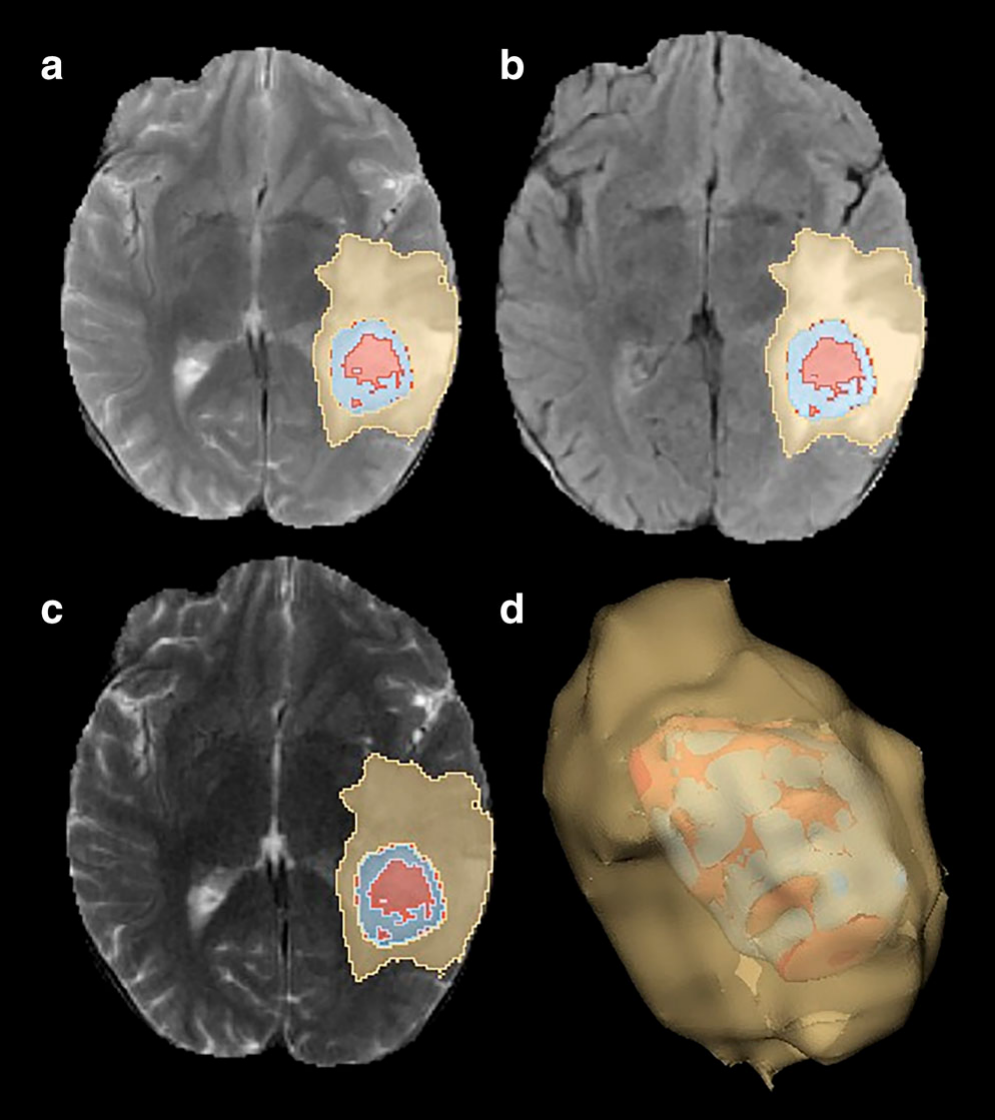
IDH wild Grade 4 Glioblastoma in the left cerebral hemisphere. (**a**) T2‐weighted images, (**b**) FLAIR‐weighted images, (**c**) T2‐FLAIR digital subtraction images, (**d**) 3‐dimensional volume rendering of the mass yellow color: perilesional edema area, blue color: contrast‐enhanced area, red: necrosis area.

**FIGURE 3:**
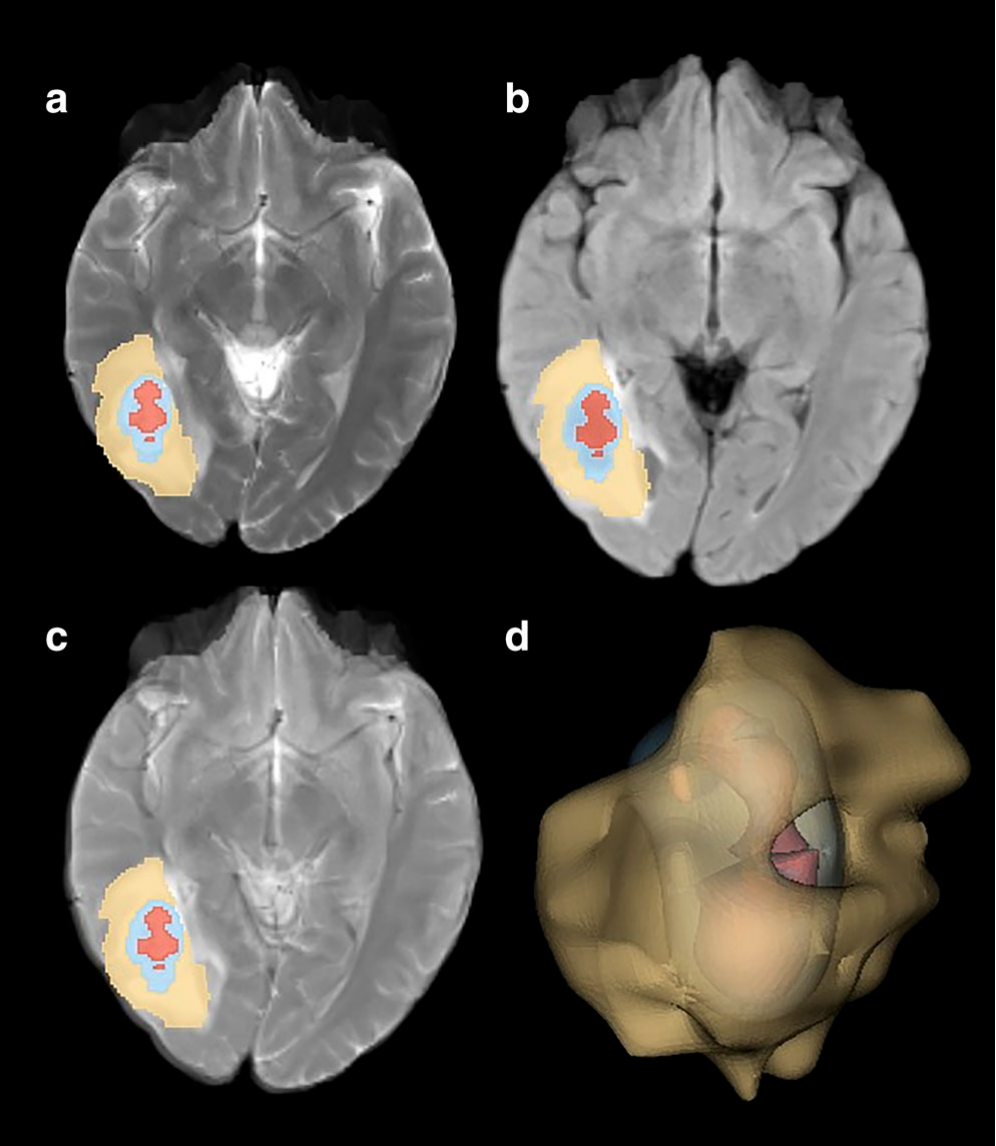
Brain metastasis from NSCLC located in the right cerebral hemisphere. (**a**) T2‐weighted images, (**b**) FLAIR‐weighted images, (**c**) T2‐FLAIR digital subtraction images, (**d**) 3‐dimensional volume rendering of the mass yellow color: perilesional edema area, blue color: contrast‐enhanced area, red: necrosis area.

Python programming language and various open‐source libraries were utilized for the three‐dimensional (3D) rendering processes. The SimpleITK library, which provides medical image processing functions, was employed for manipulating MR images and tumor masks.^25^ For the conversion into 3D rendered images, VTK (Visualization Toolkit) and matplotlib libraries, which offer 3D data processing and visualization capabilities, were utilized.^26^ Using VTK, a three‐dimensional model of each tumor region was constructed, and these models were subsequently merged. Finally, the generated 3D models were superimposed onto the MR images and visualized using matplotlib and seaborn. Illustrations of patients with IDH wild grade 4 glioblastoma (Fig. 2) and solitary brain metastases from non‐small cell lung cancer (Fig. 3) are shared.

### Extraction of Radiomics Features

Radiomic features were extracted from the peritumoral edema area on T2‐FLAIR digital subtraction images based on the PyRadiomics 3.1.0 library (http://www.radiomics.io/pyradiomics.html).^27^ Radiomic features were extracted to characterize edema encompassing the five filter types (original, Laplacian of Gaussian, logarithm, exponential, wavelet) and three feature types (first order, texture, shape) for perilesional edema area (Figs. 2 and 3). A total of 1130 features were extracted for each patient. The data for all extracted features were uploaded as Supporting Information.

### Feature Selection and Machine Learning

We used the open‐access Python 3.8 program and its open‐access libraries in all processes.^28^ Only the training cohort was used for feature selection and model development. A normalization (min–max range = 0–1) scaler was fitted on the training data and applied to both training and external validation cohorts prior to the analysis. A collinearity analysis was performed using the Pearson correlation coefficient (*r*) test, with the *r* threshold set at 0.8. Only one of the features with high collinearity was included. The training cohort was split 8:2 train/test. The maximum relevance‐minimum redundancy (mRMR) method was then used for dimensionality reduction.^29^ The Maximum Relevance Minimum Redundancy (mRMR) method is a widely recognized feature selection technique designed to identify the most informative features while minimizing redundancy among them. This method is particularly valuable in high‐dimensional data scenarios, such as radiomics, where the goal is to improve the performance of classification algorithms by selecting a subset of relevant features. The mRMR method operates on the principle of balancing two key criteria: relevance and redundancy. Relevance is measured by the mutual information between a feature and the target variable, indicating how much information the feature provides about the target. Redundancy, on the other hand, is quantified by the mutual information between pairs of features, ensuring that the selected features are not providing overlapping information. The mRMR method has shown significant success in radiomics data, particularly in binary classification tasks such as distinguishing between malignant and benign tumors.^30^ By selecting the most relevant and non‐redundant features, mRMR enhances the performance of classifiers, leading to more accurate and reliable predictions. A total of six features were used in the machine learning phase.

In our study, Naive Bayes algorithm was used to solve the classification problem. Naive Bayes is an algorithm to learn a probable machine and is based on the assumption of the Bayes theorem and “naive” (pure) independence.^31^ This assumption acknowledges that each feature is independent of the class. The Naive Bayes algorithm exhibits effective performance, especially in high‐sized data sets.^32^ The algorithm calculates the possibility distribution for each class in training data and uses these possibilities to determine which class a new example belongs to. In this study, Gaussian Naive Bayes variant was used. Gaussian Naive Bayes assumes that every feature has normal distribution when working with data constantly. During the education phase, the average and variance values were calculated for each class and classification was made using these values.

Model success was calculated in the training, test, and external validation groups belonging to centers that were never used in model development. The classifiers' performance was evaluated and compared by area under the curve (AUC). Several indices, including sensitivity (SENS), specificity (SPEC), and accuracy (ACC) were also calculated.

### Explanations of Machine Learning Model

We used SHapley Additive Explanations (SHAP) to investigate the contribution of radiomic features and the explainability of the model. SHAP allows us to better understand how the machine learning model works, and which features are influential in model decisions. SHAP values are used to measure the marginal contribution of each feature to the model output under different feature combinations, thus ensuring a fair distribution of feature importance. SHAP method makes the evaluation of feature contributions consistent and interpretable by calculating the average marginal contribution of each feature across all possible combinations.^33^ In this way, we can better understand the decision mechanism of the model, evaluate the performance of the model, and determine which features we should focus on to improve the model if necessary.

### Statistical Analysis

The data were analyzed using SPSS Statistics version 25.0 (IBM Inc. Armonk, NY, USA). Descriptive statistics were expressed as mean ± SD for continuous variables if they fit the normal distribution and median values if they do not fit the normal distribution. Mann–Whitney *U* test was used to compare continuous variables that did not show normal distribution with two‐level variables. Relationships between categorical variables were analyzed using a Chi‐square analysis/Fisher's exact test. The area under the receiver operating characteristic curve (AUC) was used to evaluate the discrimination ability of models. *P* < 0.05 was considered statistically significant.

## Results

The mean age of all patients with high grade glial tumors included in the study was 61.4 ± 13.3 years, and the mean age of all patients with metastatic brain tumors was 61.7 ± 12.2 year. Demographic data are given in Table 1.

**TABLE 1:** Demographic Data

	High‐Grade Glial Tumors (N = 788)	Brain Metastasis (N = 499)	*P*‐Value
Age (years old)	61.4 ± 13.3	61.7 ± 12.2	0.706
Sex (male)	414 (52.5%)	188 (37.7%)	0.001

**FIGURE 4:**
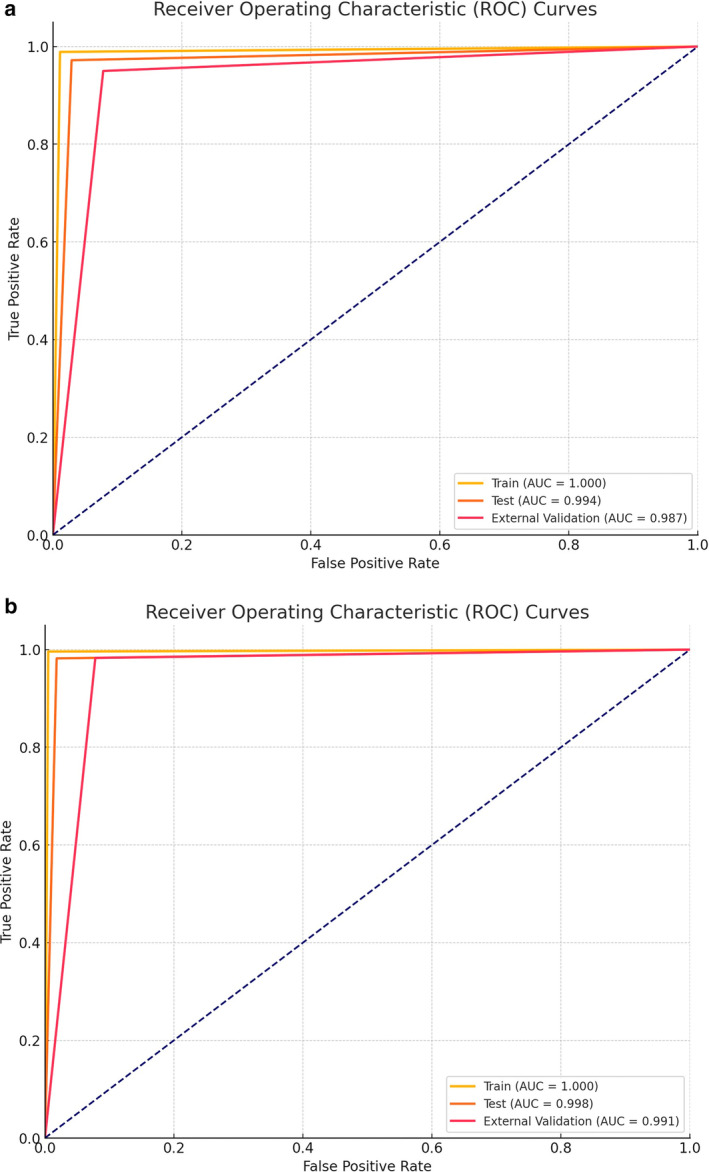
Receiver operating characteristic (ROC) curves for the model developed with patients having solitary lesions (**a**) and the model developed with all patients (**b**).

First, 702 patients with high‐grade glial tumors with solitary (single) lesions and 112 patients with solitary (single) brain metastases were evaluated, the model's AUC was 1.000 in the training group, 0.994 in the test group, and 0.987 in the external validation group. Table 2 provides a detailed evaluation of the model's success. You can examine the (receiver operating characteristic curve) ROC curve of the groups in Fig. 4a.

**TABLE 2:** Details of Model Successes in the Patient Group With Solitary (Single) Lesions

NB Model	AUC	ACC	F1	SENC	SPEC
Train (N = 662)	1.000	0.990	0.990	0.989	1.000
Test (N = 133)	0.994	0.974	0.974	0.972	0.971
External validation (N = 152)	0.987	0.967	0.970	0.950	0.922

NB = Naive Bayes; AUC = area under the curve; ACC = accuracy; F1 = F measure; SENS = sensitivity; SPEC = specificity.

Second, in patients with one or more brain metastases and in patients with one or more high‐grade primary brain tumors, differentiation between glial tumors and brain metastases, the AUC of the model's success was 1.000 in the training group, 0.998 in the test group, and 0.991 in the external validation group. Table 3 provides a detailed evaluation of the model's success. You can examine the ROC curve of the groups in Fig. 4b.

**TABLE 3:** Model Success Values Are Shown When All Brain Metastases and All High‐Grade Patients Are Evaluated

NB Model	AUC	ACC	F1	SENS	SPEC
Train (N = 804)	1.000	0.995	0.995	0.996	0.995
Test (N = 201)	0.998	0.989	0.989	0.982	1.000
External validation (N = 282)	0.991	0.951	0.951	0.983	0.922

NB = Naive Bayes; AUC = area under the curve; ACC = accuracy; F1 = F measure; SENS = sensitivity; SPE = specificity.

**FIGURE 5:**
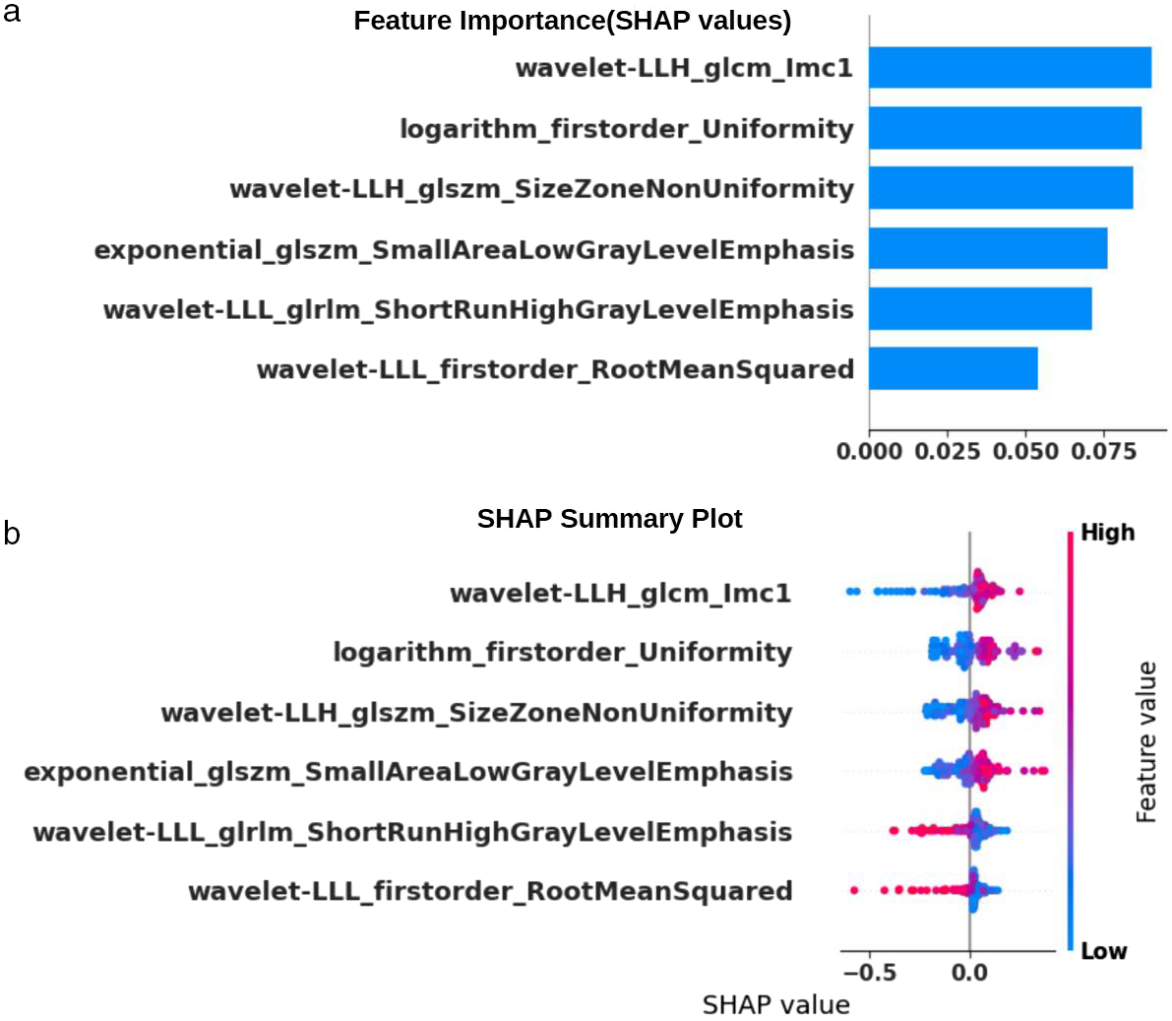
SHAP plots of the Naive Bayes model. (**a**) The classified bar charts in the SHAP summary plots illustrate the impact of each parameter on the Naive Bayes model. The graph shows that the wavelet‐LLH_glcm_lmc1 and logarithm_firstorder_Uniformity features have high SHAP values and significantly influence the model's decision. (**b**) The scatter plot in the SHAP summary plot displays the relationship between the feature values and the predicted probabilities through color coding, highlighting both positive and negative predictive effects.

**FIGURE 6:**
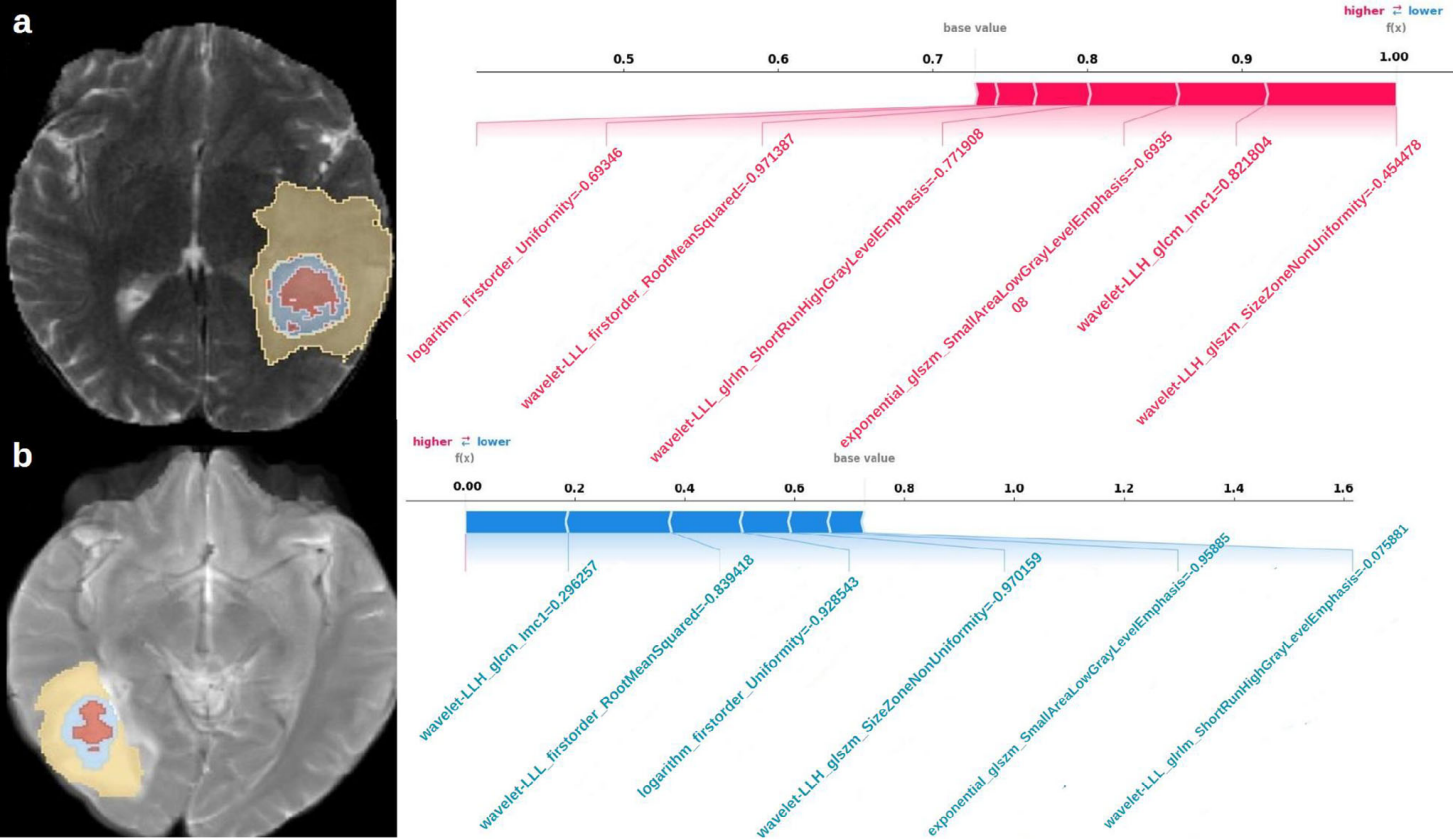
SHAP force plot interprets the evaluation of individual patients by visualizing each feature's SHAP value as a force that either increases or decreases the evaluation. Each prediction starts from the base value (0.73), which is the average SHAP value of all predictions. The length of the arrow indicates the percentage contribution of a feature's SHAP value, while the color represents whether the contribution is positive (red) or negative (blue). In (**a**), the patient's SHAP value is 0.99, higher than the base value (0.73), suggesting a diagnosis of High‐Grade Glioma (HGG). Significant positive contributions come from the wavelet‐LLH_glszm_Size‐Zone‐Non‐Uniformity feature with a value of −0.45 and the wavelet‐LLH_glcm_lmc1 feature with a value of 0.82. In (**b**), another patient's SHAP value is 0.01, lower than the base value (0.73), indicating a diagnosis of brain metastasis. For this patient, the wavelet‐LLH_glcm_lmc1 feature, with a value of 0.29, contributes negatively (blue) to the evaluation.

SHAP has provided a quantitative explanation for the Naive Bayes method. In the model developed with patients having solitary lesions, when analyzed in the external validation cohort, SHAP bar plot graphs and summary graphs visually and succinctly represented the importance range and distribution of radiomics features on the model's output, relating the feature's value to its impact. In Fig. 5a,b, we observed that the wavelet‐LLH_glcm_lmc1 feature was the radiomics feature contributing the most to the model overall. The force plot (Fig. 6) can interpret the evaluation of a single patient. It visualized a feature's SHAP value as a force that either increases or decreases the evaluation, with each prediction starting from the base value (0.73), which is the average SHAP value of all predictions. The length of the arrow explained how much (in percentage) a particular feature's SHAP value contributed. The color of the arrow represented whether the contributions were positive (red) or negative (blue). As shown in Fig. 6a, this patient's SHAP value was 0.99, which was higher than the base value (0.73), indicating that we could evaluate this patient as having HGG. Among these features, the positive (red) wavelet‐LLH_glszm_Size‐Zone‐Non‐Uniformity arrow with a value of −0.45 and the positive (red) wavelet‐LLH_glcm_lmc1 arrow with a value of 0.82 significantly contributed to the evaluation as HGG. As shown in Fig. 6b, for another patient, the SHAP value was 0.01, which was lower than the base value (0.73). Therefore, we could evaluate this patient as belonging to the BM group. In this patient, the wavelet‐LLH_glcm_lmc1 arrow, with a value of 0.29, made a negative (blue) effort to evaluate as BM.

## Discussion

Our study evaluated whether the HGG‐BM distinction could be made with machine learning models developed from radiomics data from the peritumoral edema area in T2‐FLAIR digital subtraction images. Our study detected the differential diagnosis of high‐grade glial tumor and BM with high success in patients with either solitary or multiple lesions. Model success is relatively high even in the external validation group of other centers, which was not used during the model development phase. In the SHAP analysis, when the external validation cohort was analyzed in the model developed with patients with solitary lesions, we observed that the wavelet‐LLH_glcm_lmc1 feature was the radiomics feature that contributed significantly to the model. The high success rate we achieved in our study makes us think that this could be a method that can be used in practice. In a meta‐analysis of radiomic or artificial intelligence‐based studies evaluating peritumoral edema, the success rate of differentiating high‐grade tumor from BM was reported to be 84% with the highest sensitivity and 84% specificity.^2^, ^13^, ^34^

The mechanism of peritumoral edema in brain tumors varies according to the histological type of the tumor but is mainly caused by disruption of the blood–brain barrier.^35^ Advanced imaging modalities, including diffusion‐weighted imaging and ADC, can help differentiate between brain mass lesions such as glioblastoma, brain metastases, brain abscesses, and brain lymphomas.^7^, ^36^, ^37^ However, the formation mechanism of changes in the blood–brain barrier observed around brain pathologies and contributing to the development of vasogenic brain edema differs between metastases and primary glial tumors.^35^ Some infiltrative brain pathologies, such as glioblastoma, are characterized by glial and malignant cell infiltration in the brain tissue around the tumor, and some cytokines and molecules released from these cells affect the nature of vasogenic edema.^38^ On the other hand, cytokines and molecules released by metastatic tumors and peritumoral lymphocytes are thought to be effective in the physiopathology of peritumoral vasogenic edema areas in brain metastases.^39^ Also, recent study indicate that the glymphatic system is the cause of vasogenic edema in metastases.^40^ On T2 and FLAIR‐weighted images with conventional imaging methods, peritumoral edema is often hyperintense in both high‐grade tumors and brain metastases. The peritumoral edema area contains mysterious microstructural information we do not see visually. Our study aimed to show that this microstructural structure can distinguish high‐grade glial tumors and brain metastases with pixel (radiomics)‐based information.

Previously, it has been shown that non‐enhancing gliomas using intratumoral T2‐FLAIR digital subtraction images can be used as a quantitative method to detect IDH mutant and 1p19q non‐deleted patients.^19^ The different mechanisms and pathophysiology of peritumoral edema in HGG and BMs may be the reason for the difference in our study. However, there is a need to investigate the mechanisms of peritumoral edema formation in high‐grade brain tumors and brain metastases and to examine the microstructural differences between HGG and BM. A strength of our study is that we evaluated not only the patient group with BM from a single malignancy but also the patients with multiple primary malignancies such as lung, breast, and melanoma. By including a large number of centers in our study, we created our model with images from a large number of vendors. We aimed to increase the applicability and generalizability of the model by trying to succeed in the external validation cohort of other centers that we never used during the creation and testing phase of the model. T2‐FLAIR digital subtraction images can be used for different purposes, such as subtyping in patients with brain metastases and mutation detection in patients with glial tumors.

### Limitations

Our study was retrospective. Since there were only 47 grade 4 IDH mutant astrocytoma patients in all datasets, patients with high‐grade glial tumors could be evaluated together without being separated according to IDH mutation status. Further analysis according to primary malignancy subtypes was not performed in patients with brain metastases. Other mutations that have a significant relationship with survival in high grade glial tumors could not be evaluated due to the lack of data on all patients in the retrospective structure datasets. The effect of radiomics data on survival could not be evaluated both in the metastasis group and in the HGG group.

## Conclusion

This study demonstrated that an artificial intelligence model developed with radiomics data obtained from peritumoral edema in T2‐FLAIR digital subtraction images of brain tumors might accurately distinguish between high‐grade tumors and BM. The integration of radiomics and machine learning offers a promising approach for improving the diagnostic accuracy of brain tumor classification, potentially leading to better‐informed treatment decisions and improved patient outcomes.

## Author Contributions

**Emin Demirel**: Conceptualization; methodology; software; data curation; writing – original draft. **Okan Dilek**: Formal analysis; conceptualization; writing; supervision.

## Conflict of Interest

The authors declare no conflicts of interest.

## Supporting information


**Data S1:** Supporting Information.

## References

[1] Artzi M , Bressler I , Ben BD . Differentiation between glioblastoma, brain metastasis and subtypes using radiomics analysis. J Magn Reson Imaging 2019;50(2):519‐528. 30635952 10.1002/jmri.26643

[2] Bae S , An C , Ahn SS , et al. Robust performance of deep learning for distinguishing glioblastoma from single brain metastasis using radiomic features: Model development and validation. Sci Rep 2020;10(1):12110. 32694637 10.1038/s41598-020-68980-6 PMC7374174

[3] Louis DN , Perry A , Wesseling P , et al. The 2021 WHO classification of tumors of the central nervous system: A summary. Neuro Oncol 2021;23(8):1231‐1251. 34185076 10.1093/neuonc/noab106 PMC8328013

[4] Bander ED , Jones SH , Pisapia D , et al. Tubular brain tumor biopsy improves diagnostic yield for subcortical lesions. J Neurooncol 2019;141:121‐129. 30446900 10.1007/s11060-018-03014-w

[5] Berghoff AS , Schur S , Füreder LM , et al. Descriptive statistical analysis of a real life cohort of 2419 patients with brain metastases of solid cancers. ESMO Open 2016;1(2):e000024. 27843591 10.1136/esmoopen-2015-000024 PMC5070252

[6] Fordham A‐J , Hacherl C‐C , Patel N , et al. Differentiating glioblastomas from solitary brain metastases: An update on the current literature of advanced imaging modalities. Cancer 2021;13(12):2960. 10.3390/cancers13122960 PMC8231515 34199151

[7] Byrnes TJ , Barrick TR , Bell BA , Clark CA . Diffusion tensor imaging discriminates between glioblastoma and cerebral metastases in vivo. NMR Biomed 2011;24(1):54‐60. 20665905 10.1002/nbm.1555

[8] Blanchet L , Krooshof P , Postma G , et al. Discrimination between metastasis and glioblastoma multiforme based on morphometric analysis of MR images. Am J Neuroradiol 2011;32(1):67‐73. 21051512 10.3174/ajnr.A2269 PMC7964969

[9] Lee EJ , TerBrugge K , Mikulis D , et al. Diagnostic value of peritumoral minimum apparent diffusion coefficient for differentiation of glioblastoma multiforme from solitary metastatic lesions. Am J Roentgenol 2011;196(1):71‐76. 21178049 10.2214/AJR.10.4752

[10] Price SJ , Young AM , Scotton WJ , et al. Multimodal MRI can identify perfusion and metabolic changes in the invasive margin of glioblastomas. J Magn Reson Imaging 2016;43(2):487‐494. 26140696 10.1002/jmri.24996 PMC5008200

[11] Yu H , Lou H , Zou T , et al. Applying protein‐based amide proton transfer MR imaging to distinguish solitary brain metastases from glioblastoma. Eur Radiol 2017;27:4516‐4524. 28534162 10.1007/s00330-017-4867-z PMC5744886

[12] Shin I , Kim H , Ahn S , et al. Development and validation of a deep learning–based model to distinguish glioblastoma from solitary brain metastasis using conventional MR images. Am J Neuroradiol 2021;42(5):838‐844. 33737268 10.3174/ajnr.A7003 PMC8115383

[13] Yan Q , Li F , Cui Y , et al. Discrimination between glioblastoma and solitary brain metastasis using conventional MRI and diffusion‐weighted imaging based on a deep learning algorithm. J Digit Imaging 2023;36(4):1480‐1488. 37156977 10.1007/s10278-023-00838-5 PMC10406764

[14] Gillies RJ , Kinahan PE , Hricak H . Radiomics: Images are more than pictures, they are data. Radiology 2016;278(2):563‐577. 26579733 10.1148/radiol.2015151169 PMC4734157

[15] Zhou M , Scott J , Chaudhury B , et al. Radiomics in brain tumor: Image assessment, quantitative feature descriptors, and machine‐learning approaches. Am J Neuroradiol 2018;39(2):208‐216. 28982791 10.3174/ajnr.A5391 PMC5812810

[16] Würtemberger U , Rau A , Diebold M , et al. Advanced diffusion MRI provides evidence for altered axonal microstructure and gradual peritumoral infiltration in GBM in comparison to brain metastases. Clin Neuroradiol 2024;1‐9. 10.1007/s00062-024-01416-0 PMC11339137 38683350

[17] Samani ZR , Parker D , Wolf R , Hodges W , Brem S , Verma R . Distinct tumor signatures using deep learning‐based characterization of the peritumoral microenvironment in glioblastomas and brain metastases. Sci Rep 2021;11(1):14469. 34262079 10.1038/s41598-021-93804-6 PMC8280204

[18] Patel SH , Poisson LM , Brat DJ , et al. T2–FLAIR mismatch, an imaging biomarker for IDH and 1p/19q status in lower‐grade gliomas: A TCGA/TCIA project. Clin Cancer Res 2017;23(20):6078‐6085. 28751449 10.1158/1078-0432.CCR-17-0560

[19] Cho NS , Sanvito F , Le VL , et al. Quantification of T2‐FLAIR mismatch in nonenhancing diffuse gliomas using digital subtraction. Am J Neuroradiol 2024;45(2):188‐197. 38238098 10.3174/ajnr.A8094 PMC11285991

[20] Bakas S , Sako C , Akbari H , et al. The University of Pennsylvania glioblastoma (UPenn‐GBM) cohort: Advanced MRI, clinical, genomics, & radiomics. Sci Data 2022;9(1):453. 35906241 10.1038/s41597-022-01560-7 PMC9338035

[21] Rudie JD , Saluja R , Weiss DA , et al. The University of California San Francisco Brain Metastases Stereotactic Radiosurgery (UCSF‐BMSR) MRI dataset. Radiol Artif Intell 2024;6(2):e230126. 38381038 10.1148/ryai.230126 PMC10982817

[22] Calabrese E , Villanueva‐Meyer JE , Rudie JD , et al. The University of California San Francisco preoperative diffuse glioma MRI dataset. Radiol Artif Intell 2022;4(6):e220058. 36523646 10.1148/ryai.220058 PMC9748624

[23] Scarpace L , Mikkelsen T , Cha S , et al. The cancer genome atlas glioblastoma multiforme collection (TCGA‐GBM) (version 4). The Cancer Imaging Archive2016.

[24] Clark K , Vendt B , Smith K , et al. The cancer imaging archive (TCIA): Maintaining and operating a public information repository. J Digit Imaging 2013;26:1045‐1057. 23884657 10.1007/s10278-013-9622-7 PMC3824915

[25] Yaniv Z , Lowekamp BC , Johnson HJ , Beare R . SimpleITK image‐analysis notebooks: A collaborative environment for education and reproducible research. J Digit Imaging 2018;31(3):290‐303. 29181613 10.1007/s10278-017-0037-8 PMC5959828

[26] Schroeder WJ , Avila LS , Hoffman W . Visualizing with VTK: A tutorial. IEEE Comput Graph Appl 2000;20(5):20‐27.

[27] Van Griethuysen JJ , Fedorov A , Parmar C , et al. Computational radiomics system to decode the radiographic phenotype. Cancer Res 2017;77(21):e104‐e107. 29092951 10.1158/0008-5472.CAN-17-0339 PMC5672828

[28] Abraham A , Pedregosa F , Eickenberg M , et al. Machine learning for neuroimaging with scikit‐learn. Front Neuroinform 2014;8:14. 24600388 10.3389/fninf.2014.00014 PMC3930868

[29] Peng H , Long F , Ding C . Feature selection based on mutual information criteria of max‐dependency, max‐relevance, and min‐redundancy. IEEE Trans Pattern Anal Mach Intell 2005;27(8):1226‐1238. 16119262 10.1109/TPAMI.2005.159

[30] Berrendero JR , Cuevas A , Torrecilla JL . The mRMR variable selection method: A comparative study for functional data. J Stat Comput Simul 2016;86(5):891‐907.

[31] Rish I . An empirical study of the naive Bayes classifier. Paper presented at: IJCAI 2001 workshop on empirical methods in artificial intelligence2001.

[32] Nguyen TTS , Do PMT . Classification optimization for training a large dataset with Naïve Bayes. J Comb Optim 2020;40(1):141‐169.

[33] Lundberg SM , Lee S‐I . A unified approach to interpreting model predictions. *Advances in neural information processing systems* 2017. 30.

[34] Li Y , Liu Y , Liang Y , et al. Radiomics can differentiate high‐grade glioma from brain metastasis: A systematic review and meta‐analysis. Eur Radiol 2022;32(11):8039‐8051. 35587827 10.1007/s00330-022-08828-x

[35] Solar P , Hendrych M , Barak M , Valekova H , Hermanova M , Jancalek R . Blood‐brain barrier alterations and edema formation in different brain mass lesions. Front Cell Neurosci 2022;16:922181. 35910247 10.3389/fncel.2022.922181 PMC9334679

[36] Würtemberger U , Rau A , Reisert M , et al. Differentiation of perilesional edema in glioblastomas and brain metastases: Comparison of diffusion tensor imaging, neurite orientation dispersion and density imaging and diffusion microstructure imaging. Cancer 2022;15(1):129. 10.3390/cancers15010129 PMC9817519 36612127

[37] Lemercier P , Maya SP , Patrie JT , Flors L , Leiva‐Salinas C . Gradient of apparent diffusion coefficient values in peritumoral edema helps in differentiation of glioblastoma from solitary metastatic lesions. Am J Roentgenol 2014;203(1):163‐169. 24951211 10.2214/AJR.13.11186

[38] Ostrom QT , Cioffi G , Waite K , Kruchko C , Barnholtz‐Sloan JS . CBTRUS statistical report: Primary brain and other central nervous system tumors diagnosed in the United States in 2014–2018. Neuro Oncol 2021;23(Supplement_3):iii1‐iii105. 34608945 10.1093/neuonc/noab200 PMC8491279

[39] Trembath DG , Davis ES , Rao S , et al. Brain tumor microenvironment and angiogenesis in melanoma brain metastases. Front Oncol 2021;10:604213. 33552976 10.3389/fonc.2020.604213 PMC7860978

[40] Toh CH , Siow TY , Castillo M . Peritumoral brain edema in metastases may be related to glymphatic dysfunction. Front Oncol 2021;11:725354. 34722268 10.3389/fonc.2021.725354 PMC8548359

